# Rubber Seed Oil-Based UV-Curable Polyurethane Acrylate Resins for Digital Light Processing (DLP) 3D Printing

**DOI:** 10.3390/molecules26185455

**Published:** 2021-09-08

**Authors:** Yun Hu, Guoqiang Zhu, Jinshuai Zhang, Jia Huang, Xixi Yu, Qianqian Shang, Rongrong An, Chengguo Liu, Lihong Hu, Yonghong Zhou

**Affiliations:** 1 Key Lab of Biomass Energy and Material, Jiangsu Province, Key Lab of Chemical Engineering of Forest Products, National Forestry and Grassland Administration, National Engineering Lab for Biomass Chemical Utilization, Institute of Chemical Industry of Forest Products, CAF, Nanjing 210042, China; 15150509893@139.com (Y.H.); 18354256181@163.com (G.Z.); zdslhs@126.com (J.Z.); hj18252588192@163.com (J.H.); yuxx1012@163.com (X.Y.); shang_qianqian@126.com (Q.S.); hli@icifp.cn (L.H.); zyh@icifp.cn (Y.Z.); 2 Co-Innovation Center of Efficient Processing and Utilization of Forest Resources, Jiangsu Province, Nanjing Forestry University, Nanjing 210037, China; 3 Smart Health Big Data Analysis and Location Services Engineering Lab of Jiangsu Province, Nanjing 210023, China; anrongrong@njupt.edu.cn

**Keywords:** rubber seed oil, polyurethane acrylates, UV-curable resins, high-performance, 3D printing

## Abstract

Novel UV-curable polyurethane acrylate (PUA) resins were developed from rubber seed oil (RSO). Firstly, hydroxylated rubber seed oil (HRSO) was prepared via an alcoholysis reaction of RSO with glycerol, and then HRSO was reacted with isophorone diisocyanate (IPDI) and hydroxyethyl acrylate (HEA) to produce the RSO-based PUA (RSO-PUA) oligomer. FT-IR and ^1^H NMR spectra collectively revealed that the obtained RSO-PUA was successfully synthesized, and the calculated C=C functionality of oligomer was 2.27 per fatty acid. Subsequently, a series of UV-curable resins were prepared and their ultimate properties, as well as UV-curing kinetics, were investigated. Notably, the UV-cured materials with 40% trimethylolpropane triacrylate (TMPTA) displayed a tensile strength of 11.7 MPa, an adhesion of 2 grade, a pencil hardness of 3H, a flexibility of 2 mm, and a glass transition temperature up to 109.4 °C. Finally, the optimal resin was used for digital light processing (DLP) 3D printing. The critical exposure energy of RSO-PUA (15.20 mJ/cm^2^) was lower than a commercial resin. In general, this work offered a simple method to prepare woody plant oil-based high-performance PUA resins that could be applied in the 3D printing industry.

## 1. Introduction

Ultraviolet (UV)-curable technology is an instantaneous and effective method for converting liquid ingredients into a crosslinked solid polymer network [1]. UV-curable materials have gained widespread attention for the advantages of high curing efficiency, low energy consumption, low solvent emission, low costs, and moderate curing condition. They are widely used in the fields of coatings, adhesives, and high value-added products [2,3]. However, most of the UV-curable raw materials were prepared from petrochemical products. One major drawback of them lies in the excessive dependence on the upstream supply chains, which leads to the unstable supply of raw materials and high prices. Besides, petrochemical products are non-renewable, which causes more pressure on environmental supervision [4]. For the purposes of reducing the consumption of fossil feedstock and endowing materials with biocompatible and biodegradable properties, various resources of renewable building blocks [5,6,7], including plant oil [8,9,10,11], natural phenolic [12,13,14,15,16,17], natural acid [2,18,19,20,21], and so forth [1,22,23,24], have been employed as starting materials to prepare UV-curable materials [25].

As one of the most common UV-curable resins, polyurethane acrylate (PUA) has attracted much attention in UV-curable industry attributing to its excellent flexibility, prominent adhesion and a variety of adjustable features [26,27]. However, PUA resins generally exhibit low tensile strength and slow UV-curing speed for insufficient crosslink density, which limits their practical applications in some fields [28]. Therefore, various works regarding improving the properties of PUA’s mechanical properties, as well as coating properties, have been reported. In Li’s [29] work, castor oil/pentaerythritol triacrylate-based waterborne polyurethane acrylate (WPUA) was developed and mechanical property performance of the cured WPUA was improved, which related to the formation of the compact cross-linked structure. Wei et al. [28] reported a series of castor oil-based UV-curable PUA resins via a thiol-ene click chemistry. The resultant cured films exhibited high acrylate double-bond conversion and excellent flexibility and water resistance. Our group obtained [30,31] biobased polyfunctional PUA oligomers in the presence of pentaerythritol triacrylate with a high performance, and the relationship between structure and performance, as well as the UV-curing behaviors of the resultant bioresins, were investigated systematically. Although the promotion of functionality in above-mentioned methods has been achieved, the disadvantage of high molecular weight emerged during the research of such PUAs, which led to a high viscosity for the UV curable materials. The utilization of fatty acids in polyol synthesis gave narrower molecular weight distribution compared to a mixture of fatty acids in the triglyceride structure. Paraskar et al. [32] designed and synthesized PUA oligomers by utilizing renewable dimer fatty acids. The resulting biomass coatings exhibited exceptional results, including the thermal degradation temperature of 382–422 °C, glass transition temperature of 56.38–66.27 °C, and the tensile strength of 17.3–30.6 MPa. These results indicated that biobased UV-curable PUA materials exhibited great potential to replace the petroleum-based ones.

Plant oils have been extensively utilized to develop oil-based PUA materials owing to environmental friendliness, low price, biodegradability, and sufficient source. Woody plants oil, such as rubber seed oil, are preferrable for the reason that they don’t compete with agricultural food plants for farmland [33]. In our previous work, we acquired hydroxylated woody plant oils via epoxidation and hydroxylation reactions, and then synthesized polyfunctionality oil-based PUA oligomers, and finally produced high-performance woody plant-oils-based UV-cured materials [34]. However, the synthetic procedures for the hydroxylated woody plant oil precursors were complicated, and the oligomers tended to gel at room temperature due to the high amount of carbon–carbon double bonds. To overcome the above obstacles, a facile method via the alcoholysis of rubber seed oil (RSO) was utilized to synthesize hydroxylated RSO (HRSO). The resulting HRSO reacted with the hydroxyethyl acrylate (HEA)-modified IPDI and the high-functional RSO-based PUA (RSO-PUA) oligomer was synthesized. Finally, diluents were employed to co-photopolymerize with RSO-PUA and the ultimate properties of the resultant materials, as well as the UV-curable behavior of the bioresin, were carefully studied, and the DLP 3D printability of the optimal resin was also demonstrated.

## 2. Materials and Methods

### 2.1. Materials

Rubber seed oil was obtained from Southwest Forestry University Academy of Forestry. From Sahn Chemical Technology Co., Ltd. (Shanghai, China), the following were purchased: 2-Hydroxy-2-methylpropiophenone (Darocur 1173, 98%), 4-methoxyphenol (98%), triethylene glycol diacrylate (TEGDA, 98%), and trimethylolpropane triacrylate (TMPTA, 99%). Glycerol was obtained from Nanjing Chemical Reagent Co., Ltd. (Nanjing, China). Dibutyltin dilaurate (DBTDL, ≥95%) was provided from Shanghai Titan Scientific Co., Ltd. (Shanghai, China). Isophorone diisocyanate (IPDI, 99%) was purchased from Jiuding Chemistry Co., Ltd. (Jiaxing, China). Hydroxyethyl acrylate (HEA, ≥97%) obtained from Macklin Chemical Reagent Co., Ltd. (Shanghai, China). Commercial resin was purchased from Shenzhen Creality 3D Technology Corporation (Shenzhen, China). All of the solvents were used as received without further purification.

### 2.2. Synthesis of HRSO

**Scheme 1:**
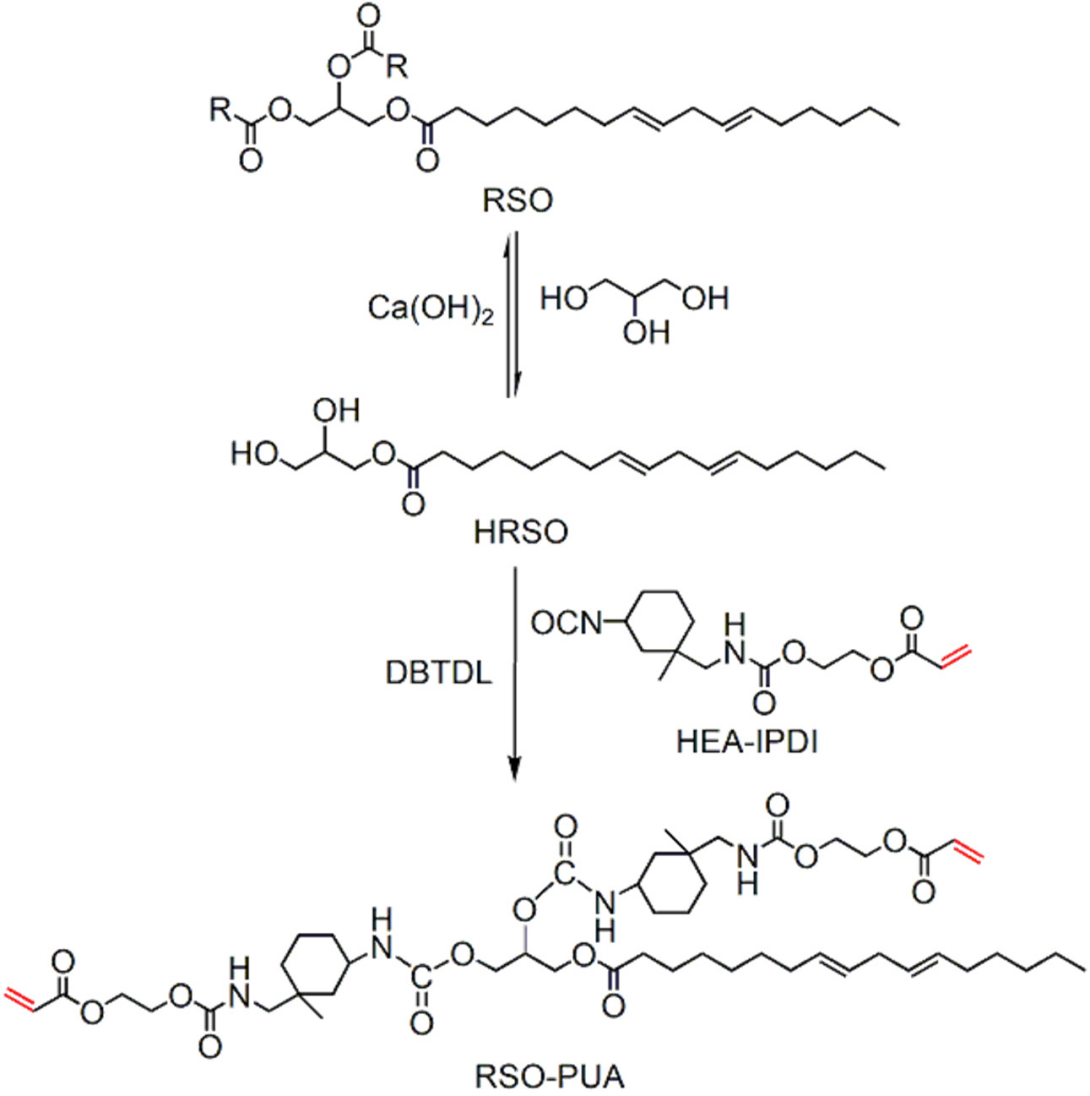
Synthesis route for RSO-PUA.

The hydroxylation of RSO with glycerol was carried out to produce HRSO, based on previous works [35,36]. About 255.9 g (0.30 mol) of RSO, 69.3 g (0.75 mol) of glycerol, and 3.3 g of Ca(OH)_2_ were placed together in a 500 mL four-neck round-bottom flask equipped with a mechanical stirrer, a thermometer, a nitrogen (N_2_) gas inlet, and a refluxing condenser with a calcium drier. The flask was placed in a heating mantle with a temperature controller. The reaction mixture was heated to about 160 °C for 20 min under N_2_, and 220 °C for 2 h at this temperature. At the end of the reaction, the product was cooled rapidly by ice water bath to atmospheric temperature. The obtained product of HRSO was a brown paste at room temperature (Scheme 1).

### 2.3. Synthesis of RSO-PUA

Typically, 44.6 g (0.2 mol) of IPDI, normal hexane, 0.3 g of 4-methoxyphenol and 0.9 g of DBTDL were heated to 50 °C in a four-necked flask with a mechanical stirrer and thermometer under N_2_. 23.2 g of (0.2 mol). HEA was slowly dropped into the flask at 50 °C for 4 h and obtained the prepolymer hydroxyethyl acrylate-modified isophorone diisocyanate (HEA-IPDI). Next, HRSO was slowly added to HEA-IPDI and heated to 50 °C for 5 h. Finally, the product RSO-PUA was light yellow, transparent liquid resin at room temperature.

### 2.4. Curing of RSO-PUA Resins

As shown in Table 1, the UV-curable samples were obtained by blending the RSO-PUA diluents (the chemical structures were shown in Figure S1) and photoinitiator 1173 for 20 min at room temperature. The mixtures were degassed in vacuum dryer for about 10 min. The resulting samples were then cast into homemade polytetrafluoroethylene molds or coated on polished tinplate sheets by a film maker. Finally, the cast or coated samples were cured by a UV light-curing microprocessor (Intelli-Ray 400 W, Uvitron International Corporation, Springfield, MA, USA). The exposure intensity and time for all the samples was 100 mW/cm^2^ and 5 min, respectively.

### 2.5. Digital Light Processing (DLP) Three-Dimensional (3D) Printing

The model was designed in ProE 3.0 (Parametric Technology Corporation, Boston, MA, USA) and printed by the DLP 3D printing method. A custom-built microlithography system with a 405 nm UV-light, was used to cure the RSO-PUA resin layer by layer. The football-ene structure was printed using a LD-002H 3D-printer (Shenzhen Creality 3D Technology Corporation, Shenzhen, China). The exposure time for each layer was set to 8–10 s. After printing, the surface of obtained football-ene model was washed with ethanol, and the structure was postcured by the Intelli-Ray 400W UV light-curing microprocessor with an exposure intensity of 100 mW/cm^2^ for 10 min.

### 2.6. Characterization

FT-IR spectra of samples were conducted using a Nicolet iS10 IR spectrometer (Thermo-Fisher, WalthaM, MA, USA) with a scanning range from 4000 to 500 cm^−1^. ^1^H NMR spectra of samples were recorded using a DRX-300 spectrometer (Bruker, Germany) with CDCl_3_.

Gel content (*C*_gel_) of the UV-cured samples was measured via Soxhlet extraction. Typically, the specimen with a weight of approximately 0.5 g was weighted, wrapped in a filter paper, and refluxed for 24 h. Finally the specimen was dried at 60 °C until a constant mass was obtained. The *C*_gel_ data was calculated from the mass ratio of the UV-cured specimen before and after extraction.

The fractured surfaces of the UV-cured resins were studied with a Hitachi 3400e1 (Hitachi Instrument Corporation, Tokyo, Japan) scanning electron microscope (SEM) instrument operated at 12 kV. The fractured films were stained on a copper grid with a conductive adhesive. The cross sections were coated with gold prior to SEM observation. Dynamic mechanical analysis (DMA) of the UV-cured samples was conducted on a Q800 solids analyzer (TA Corporation, Westlake, OH, USA) within a film-tension mode with an oscillating frequency of 1 Hz. All the samples with a size of 40 × 6 × 1 mm^3^ were swept from −80 °C to 200 °C and a heating rate of 3 °C/min.

Thermogravimetric analysis (TGA) of the UV-cured samples was performed on an STA 409PC thermogravimetry instrument (Netzsch Corporation, Selb, Germany). The samples were heated from 40 to 800 °C under nitrogen gas at a flow rate of 100 mL/min and at a heating rate of 10 °C/min.

Mechanical properties of the UV-cured samples were tested using a SANS7 CMT-4304 Universal tester (Shenzhen Xinsansi Jiliang Instrument Corporation, Shenzhen, China) with a cross-head speed of 5 mm min^−1^. The dimension of all specimens was 60 × 10 × 1 mm^3^. The calculated values were determined from an average value of five specimens.

Coating properties of the UV-cured coatings including adhesion, pencil hardness, and flexibility were measured in GB 1720-79(89), GB/T 6739-2006, and GB/T 6739-1996, respectively. Detailed procedures for the coating tests were reported in our previous works [37].

Swelling properties of the UV-cured samples were determined including water, ethanol, acetone, and toluene. Specimens were weighed precisely and immersed into various solvents at atmospheric temperature for 48 h. Then they were dried and weighed again. Swelling degree of the specimens was calculated through the following equation:

$$S=\frac{W_{1}-W_{0}}{W_{1}}\times 100\%$$

where *W*_0_ and *W*_1_ were the weight of the sample before and after immersion in solvents, respectively.

UV-curing behaviors of the resultant liquid resins were investigated by a modified Nicolet 5700 spectrometer (Thermo-Nicolet Instrument, Waltham, MA, USA). The conversion of carbon–carbon double bond (*α*_C=C_) was determined by monitoring the change of absorption at about 810 cm^−1^, which was calculated according to the following formula:

$$\alpha_{C=C}=\frac{A_{0}-A_{t}}{A_{t}}\times 100\%$$

where *A*_0_ and *A*_t_ were the C=C peak areas at the original time and t time, respectively.

## 3. Result and Discussion

### 3.1. Structural Characterization of RSO-PUA Oligomer

**Figure 1:**
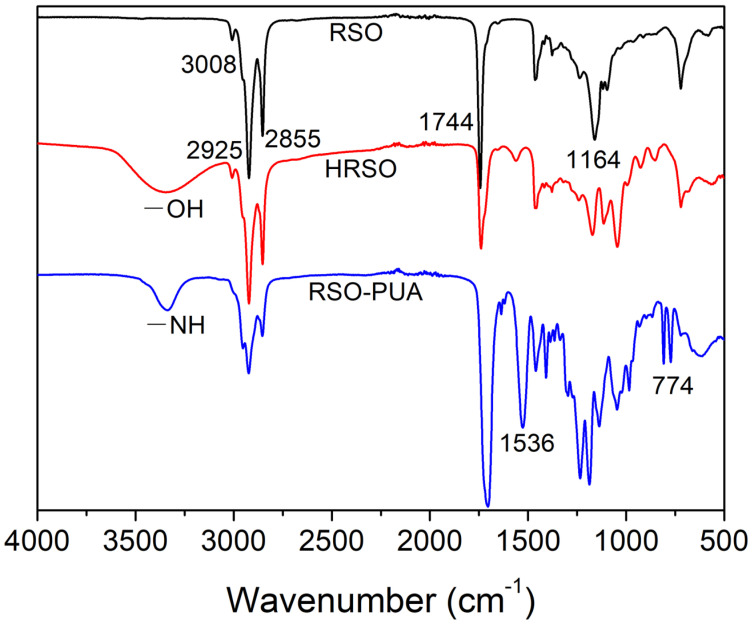
FT-IR spectra of RSO, HRSO and RSO-PUA.

The chemical structures of the RSO-based intermediate and final product were characterized by FT-IR and ^1^H NMR. The FT-IR spectra of RSO, HRSO, and RSO-PUA are depicted in Figure 1. The FT-IR spectrum of IPDI-HEA is shown in Figure S2. In the spectrum of RSO, the characteristic peak at 3008 cm^−1^ was derived from the stretching vibration band of =C–H [38]. The peak occurred at 2931 cm^−1^ and 2854 cm^−1^, which arose from the C–H asymmetric stretch vibration in methylene and methyl group stretching on the chain, the strong peak at 1744 cm^−1^ was ascribed to the C=O groups of triglyceride [10]. Comparing the spectrum of HRSO with that of RSO, a broadband appeared at 3380 cm^−1^ for hydroxyl stretching frequency, the stretching vibration peak of C–O– at 1164 cm^−1^ was weakened. The variation indicated that the RSO was successfully hydroxylated with glycerol. In the spectrum of RSO-PUA, the peak at 3380 cm^−1^ corresponding to hydroxyl vanished, a new peak at 3363 cm^−1^ attributed to the –NH bonds of carbamate was observed; the peaks at 1536 cm^−1^ and 774 cm^−1^ were attributed to urea stretching C–N bending and N–H vibration, respectively. Moreover, the absorption peak at 2265 cm^−1^ was not observed in the spectrum of RSO-PUA, which represented the –NCO vibrations from IPDI [39]. All the changes indicated that HRSO was successfully grafted with HEA-IPDI.

**Figure 2:**
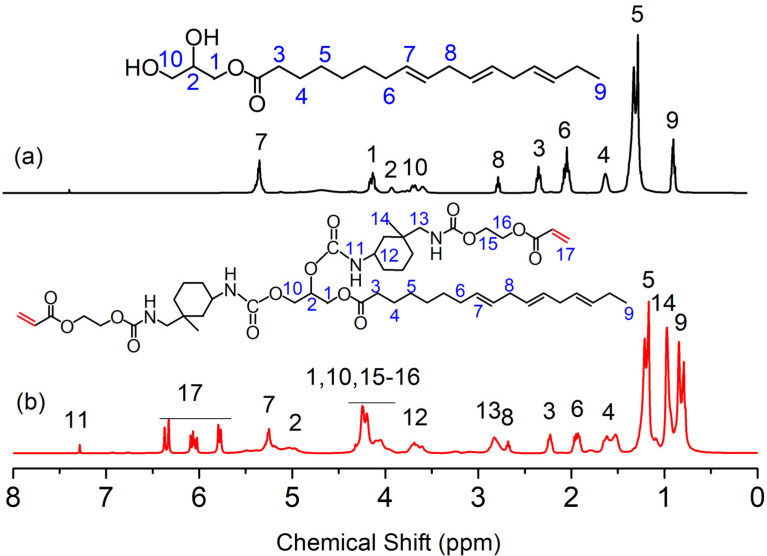
^1^H NMR spectra of (**a**) HRSO and (**b**) RSO-PUA.

^1^H NMR spectra of HRSO and RSO-PUA are presented in Figure 2. The ^1^H NMR spectrum of RSO is shown in Figure S2. In spectrum of HRSO, the peaks at 3.4–4.0 ppm were attributed to –CH_2_– and –CH– protons of glycerol group. In the spectrum of RSO-PUA, the peaks at 5.7–6.4 ppm were attributed to the –C=CH_2_– protons of the acrylate group [35]. The peaks which appeared at 3.8–4.3 ppm represented the protons of glycerol moiety and methylene of IPDI-HEA; the peaks which occurred at 2.7–2.8 and 3.5−3.7 ppm were assigned to the protons linked to carbamates. The appearance of all chemical shifts indicated that RSO−PUA was successfully synthesized. For plant oil triglycerides, the peak at 2.3 ppm, corresponding to the methylene protons attached to the ester group, could be taken as reference, since the intensity of the peak should not change throughout all the reactions. The peaks at 5.7–6.5 ppm, corresponding to the protons of the C=C groups, were all from HEA. Therefore, the introduced *α*_C=C_ of the RSO−PUA resin was estimated by ^1^H NMR and could be determined by the following equation [40]:

$$\alpha_{C=C}=\frac{A_{5.7-6.5ppm}/3}{A_{2.3ppm}/2}=\frac{2A_{5.7-6.5ppm}}{3A_{2.3ppm}}$$

The introduced *α*_C=C_ of RSO-PUA product was 2.27 per fatty acid, which was equal to 6.81 per triglyceride.

### 3.2. Gel Contents

The *C*_gel_ is the insoluble fraction representing the degree of cross-linking of the UV-cured RSO-PUA films, and the data was assessed with the aid of Soxhlet extraction [41]. The values of *C*_gel_ are listed in Table 1. As the content of HEA increased from 0 to 40, the *C*_gel_ of RSO-PUA/HEA samples rose from 83.9% up to 93.0%, which indicated that the addition of diluent promoted the degree of cross-linking. Additionally, the samples cured with tri-functional diluent (TEGDA) and di-functional (TMPTA) diluent exhibited higher gel content than mono-functional diluents (HEA), ranked by the order RSO-PUA/40% TMPTA (98.5%) > RSO-PUA/40% TEGDA (95.0%) > RSO-PUA/40% HEA (93.0%), indicating that the high-functionality diluents could enhance the degree of cross-linking of the UV-cured samples [32].

### 3.3. Properties of the UV-Cured RSO-PUA Materials

#### 3.3.1. Dynamic Mechanical Analysis

**Figure 3:**
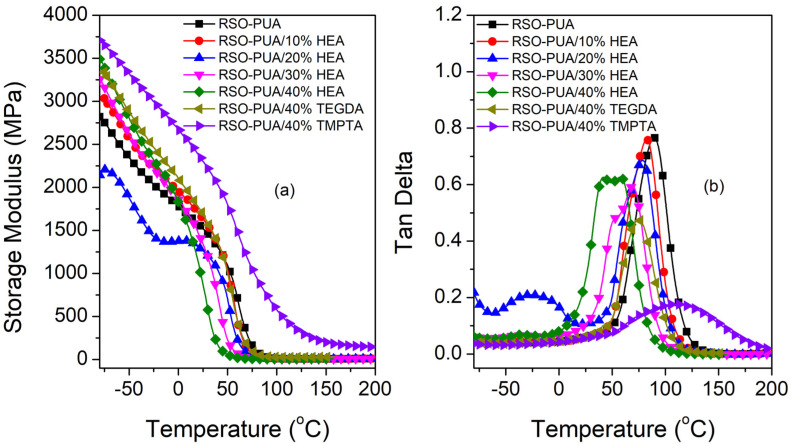
(**a**) Storage modulus and (**b**) loss factor versus the temperature of the UV-cured RSO-PUA materials.

DMA technique was employed to investigate the thermal–mechanical properties of the prepared resins. The curves of tan *δ* and the storage modulus of the UV-cured RSO-PUA materials as a function of temperature are shown in Figure 3. The corresponding data, such as storage modulus (*E′*), glass transition temperature (*T*_g_), and cross-link density (*ν*_e_) are summarized in Table 2. As can be seen in Figure 3, high *E′* values of all samples were observed in the glassy state, but quickly decreased in the high-temperature region, and eventually approached 0 MPa in a rubbery state. As the content of HEA growing, the storage modulus at 25 °C (*E′*_25_) of the RSO-PUA/HEA materials changed from 1.5 GPa to 0.9 GPa; the reason is that the addition of diluent weakened the physical crosslinking density of the cured films through the intermolecular forces of hydrogen bonding (urethane groups) [10]. The *T*_g_ showed a similar variation trend, and RSO-PUA/40% TMPTA exhibited the highest *T*_g_ of 109.4 °C, due to its highest cross-link density, which was calculated below. Moreover, the tan δ peak became less intensive and broader with the increased diluent, due to the less uniform cross-linked network structure in the cured materials. *T*_g_ was obtained from peak temperatures of the tan *δ* curves, and *ν*_e_ was determined according to the following equation [2,42]:

$$\nu_{e}=\frac{E^{\prime }}{3RT}$$

where *E′* is the storage modulus in the rubbery plateau region (the *E′* at the temperature of *T*_g_ was employed for the calculation of *ν*_e_), *R* represents the universal gas constant, and *T* denotes the absolute temperature. As shown in Table 2, as the content of HEA increased from 0 to 40%, the cured resins exhibited a high *ν*_e_ of above 100 × 10^3^ mol/m^3^. Owing to the more carbon–carbon double bonds introduced by polyfunctionality diluent [11], the RSO-PUA/40% TEGDA and RSO-PUA/40% TMPTA resins showed a higher *ν*_e_ than the RSO-PUA/HEA series, especially for the RSO-PUA/40% TMPTA revealing the highest *ν*_e_ (241.2 × 10^3^ mol/m^3^) among the cured materials.

#### 3.3.2. Thermogravimetric Analysis

**Figure 4:**
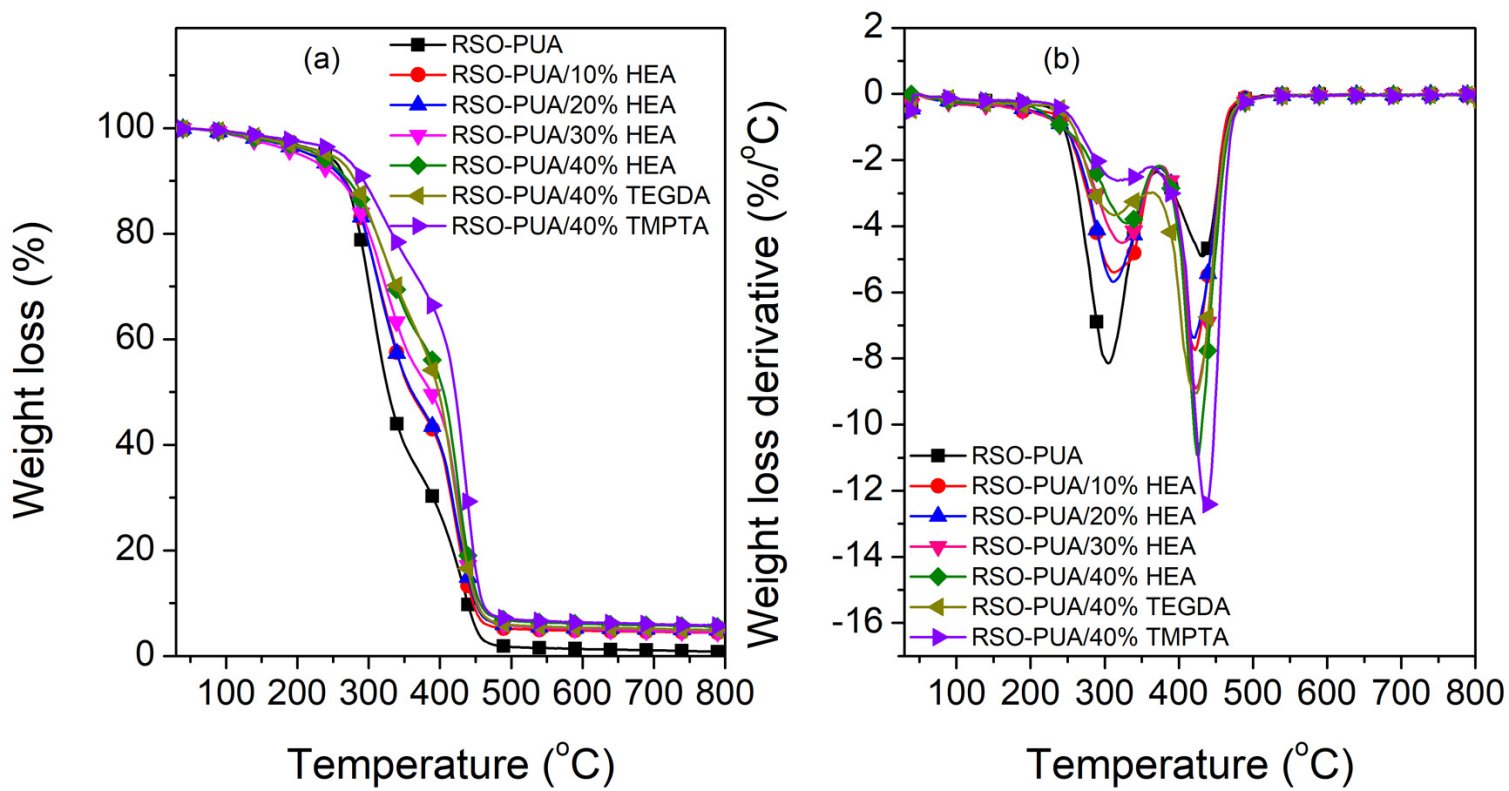
(**a**) TG and (**b**) DTG of the UV-cured RSO-PUA materials.

The TGA and their corresponding derivative weight loss (DTG) of the developed RSO-PUA samples under the N_2_ atmosphere are plotted in Figure 4, and related data of the 5% weight loss temperature (*T*_5%_), 10% weight loss (*T*_10%_) and maximum decomposition temperature of weight loss rate (*T*_max_) are listed in Table 2. The cured materials of RSO-PUA/HEA displayed inferior thermal stability compared with that of RSO-PUA/40% TEGDA and RSO-PUA/40% TMPTA cured samples. The reason probably lies in that RSO-PUA/HEA-cured materials possessed lower crosslink densities, which were calculated in DMA [11]. The decomposition of the RSO-PUA materials consisted of two phases as indicated by the appearance of two main weight-loss regions with peak maxima around 300–330 °C and 410–440 °C in the DTG curves, respectively. First, the weight loss below 270 °C can be attributed to the removal of solvent and moisture and the decomposition of micromolecules [43]. Second, the decomposition stage corresponded to the hard segments breakage of urethane linkage. Finally, the step above 500 °C can be assigned to the slow degradation of soft segments decomposing and char residue. In short, all UV-cured resins showed high thermal stability, suggesting that they had the potential to be used as UV-curable coatings for relative high-temperature-resistance substrates.

#### 3.3.3. Mechanical Properties

**Figure 5:**
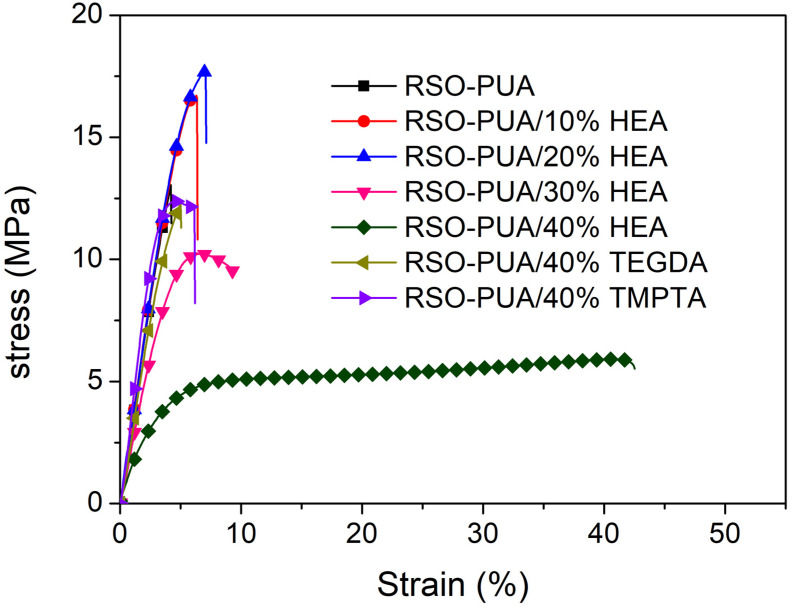
Tensile stress−strain curves of the UV-cured RSO-PUA resins.

The stress–strain curves of RSO-PUA samples are depicted in Figure 5, and detailed data is summarized in Table 3. Figure S5 presents the comparison of RSO-PUA/40%HEA resin and commercial resin, and the corresponding data are listed in Table S1. As the content of HEA increased from 10 to 40%, the tensile strength and tensile modulus of the cured RSO-PUA/HEA materials dropped from the maximum values of 16.3 MPa and 330.6 MPa to 5.4 MPa and 113.3 MPa, respectively. The decreased *ν*_e_ (showed by the DMA above) most likely accounts for the variation in ductility and stiffness [41]. The tensile strength of the pure RSO-PUA sample was similar to that of RSO-PUA with polyfunctionality diluent (TEGDA and TMPTA), which may due to the carbon–carbon double bond functionality of the RSO-PUA oligomer (2.27 per aliphatic acid) and polyfunctional diluent was approximate. Overall, the RSO-PUA/10%HEA and RSO-PUA/20%HEA film achieved the best mechanical property among all samples with approximately 16 MPa in tensile strength and 7% strain in elongation at break.

#### 3.3.4. Coating Properties

The surface properties such as the pencil hardness, flexibility and adhesion of the obtained RSO-PUA coatings were measured and the related results are summarized in Table 3. With the growth of HEA from 0 to 40%, the adhesion and flexibility maintained unchanged at the superior values of 2 mm and 2 grade, and the pencil hardness dropped from 2H to H and then grew to 3H. The variation of hardness is possibly attributed to the changed values of *ν*_e_ [35]. Moreover, as the C=C functionality of diluent increased from 1 to 3, the pencil hardness maintained unchanged at 3H, the adhesion and flexibility still demonstrated at 2 grade and 2 mm. Despite this, the RSO-PUA/40% TEGDA films displayed an inferior adhesion and flexibility, which could also be ascribed to the decrease in hydroxyl concentration compared with HEA [40].

#### 3.3.5. Fractured Surface Morphology Analysis

A morphological study was assessed using SEM, and the corresponding fractured surface SEM images of the UV-cured RSO-PUA materials are illustrated in Figure S4. Ductile fracture allowed the cracks or defects to propagate in different directions, which gave rough cleavage. In the brittle fracture, the polymer chains in structure glided on each other, which resulted in smooth cleavage. As the HEA content increased from 0 to 30%, the RSO-PUA/HEA resin had a higher area associated with ductile fracture, where the roughness could be seen in the image. However, the RSO-PUA resins with 40% diluent (HEA, TEGDA, TMPTA) had a higher fraction of the brittle fracture and a slight ductile fracture region, where the flake type of morphology with a short, smooth area was observed.

#### 3.3.6. Swelling Properties

The swelling properties of the UV-cured RSO-PUA materials were investigated and the corresponding results were summarized in Table 4. Firstly, the water absorption of all the UV-cured RSO-PUA coatings was below 0.5%, which can be ascribed to the higher *ν*_e_. As we know, the more compact network of coatings resulted in smaller porous volume [43]. Second, the obtained materials exhibited better resistance to water than to organic solvents (ethanol, acetone, and toluene). Furthermore, as the HEA content increased from 0 to 40%, the absorbed amount of water, and organic solvent did not show a clear decreasing trend, while the above values for both the polyfunctional diluent systems were clearly lower than that of monofunctional diluent, especially for the RSO-PUA/40% TMPTA materials. That was because TMPTA possessed the analogous swelling characteristic to the RSO-PUA oligomer and possessed highest *ν*_e_.

### 3.4. UV-Curing Kinetics of the RSO-PUA Resins

**Figure 6:**
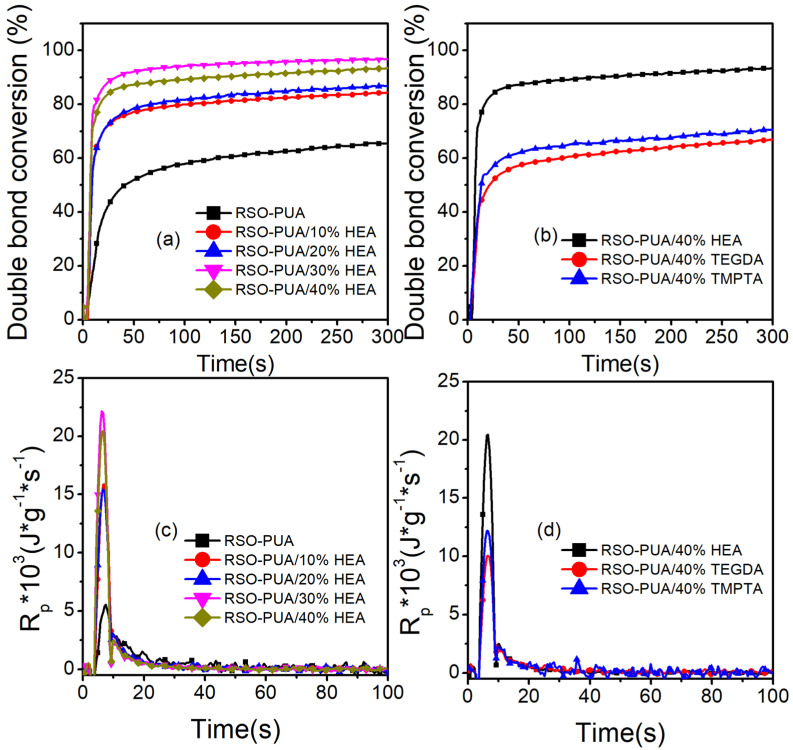
(**a**,**b**) Double bond conversions and (**c**,**d**) polymerization rate of the RSO-PUA resins.

As shown in Figure 6, the UV-curing behaviors of RSO-PUA materials were performed with RT-IR, and the related data were presented in Table 4. As the HEA content rose from 0 to 40%, both the C=C conversions (α_f_) and maximum C=C conversion rate (R_p_) of RSO-PUA/HEA resins increased from 65.5% to 97.4% and 5.5 s^−1^ to 22.2 s^−1^, respectively, apart from the values of RSO-PUA/40% HEA, which dropped slightly to 93.1% and 20.5 s^−1^, respectively. The variation possibly lies in that the increased dosage of HEA decreased the viscosity of the RSO-PUA resins and increased the colliding chance for C=C groups, while excess HEA can be homopolymerized during the co-polymerization of RSO-PUA with HEA, leading to a decreased HEA concentration for the RSO-PUA diluting and co-polymerization [44]. Besides, when the C=C functionality of the diluent increased from 1 to 3, the α_f_ and R_p_ exhibited the declined trend for the polyfunctional diluent, meaning that the use of the diluent with the higher functionality would hamper the α_f_ and R_p_ of the RSO-PUA resins [40]. In addition, the R_p_ values for all the resins reached a maximum value within <10 s, suggesting that the polymerization rate was excellent. The high-functionality of the developed RSO-PUA oligomer may account for the excellent polymerization rate.

### 3.5. 3D Printing Performance

**Figure 7:**
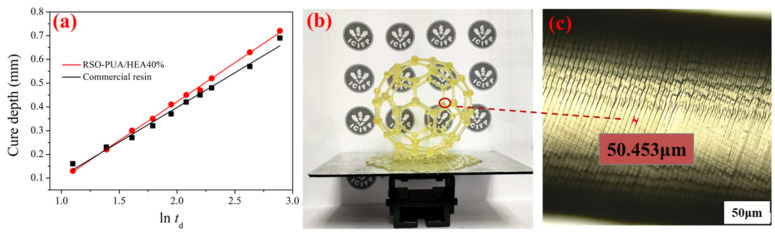
(**a**) Working curves of the UV-curable RSO-PUA/HEA40% resin and commercial resin; (**b**) the printed model of football-ene; (**c**) the surface image of the football-ene model.

The parameters of penetration depth (*D*_p_) and the threshold dose of energy (*E*_c_) were employed to assess the curing properties of UV-curing printing resins [44]. The two values could be calculated according to the following equations derived from the Beer–Lambert law [45,46]:

$$Z_{d}=D_{p}\ln\frac{t_{d}}{t_{0}}\;E_{c}=I_{0}t_{0}$$

where *Z_d_* is the cure depth, *t*_0_ is the critical cure time, *t_d_* is the exposure time, and *I*_0_ is the intensity at *Z_d_* = 0. By measuring and plotting the cure depth of the resin as a function of the different exposure times, the data of *D*_p_ and *t*_0_ was obtained from the slope and the intercept of the fitted line, respectively [46]. As with previous reports, the viscosity of a polymer solution higher than 3 Pa·s was not suitable for DLP-based 3D printing [3]. Combined with the overall properties and viscosity of the RSO-PUA resin, the RSO-PUA/HEA40% was selected as the print model. The fitted working curves are shown in Figure 7a, and the detailed data are listed in Table 5. The *D*_p_ value of RSO-PUA/HEA40% resin was 0.327 mm, which was comparable to that of DLP-based 3D printing commercial resin. To reduce the *D*_p_, the UV-photo absorber was usually added to the commercial resin and lower *D*_p_ is related to higher printing resolution. Besides, the *E*_c_ data of RSO-PUA/HEA40% was lower 1.12 mJ/cm^2^ than that of commercial resin [47,48], which was likely due to the high functionality of the RSO-PUA oligomer [48]. Thus, the *D*_p_ and *E*_c_ values of RSO-PUA/HEA40% were suitable for general DLP-based 3D printing.

An object of football-ene was printed by the DLP-3D printer, and the surface of the football-ene was measured using an optical microscope to observe the layer thickness. As shown in Figure 7c, the layer thickness of DLP-3D printing RSO-PUA/HEA40% resin was 50.453 µm, which was very close to the setting value (50 µm) by the slice software. In addition, the surface of the model showed no obvious cracks or gapes between the layers, meaning the excellent adhesion between layers. Therefore, the printed model with RSO-PUA was available for general printing.

## 4. Conclusions

In this work, the novel RSO-based PUA oligomer was first synthesized and then a series of UV-curable resins were prepared by blending the pure RSO-PUA with various diluents. Properties of the cured materials involving thermal properties, dynamical mechanical properties, mechanical properties, coating performance, and solvent resistance, were studied. The obtained UV-cured RSO-PUA resins demonstrated a superior gel content (up to 98.5%), high *T*_g_ (up to 109.4 °C), excellent thermal stabilities (*T*_5%_ up to 270 °C), and a high mechanical strength (up to 16.3 MPa). The high content of diluent and the increase of diluent functionality were beneficial to the gel content, *T*_g_ and thermal stabilities of the UV-cured resins. Additionally, the cured resins exhibited excellent hardness and flexibility, strong adhesion, and remarkable water resistance. The optimal resin showed lower *E*_c_ than the commercial resin and the football-ene model was successfully printed by DLP 3D printing. In summary, this work provides a facile strategy to fabricate woody plant oil-based UV-curable PUA resin for DLP 3D printing.

## Figures and Tables

**Table 1:** Composition and gel content for the UV-curable RSO-PUA samples.

Samples	RSO-PUA (%)	HEA (%)	TEGDA (%)	TMPTA (%)	D1173 (%)	Gel Content (%)
RSO-PUA	97	0	0	0	3	83.9 ± 0.9
RSO-PUA/10% HEA	87	10	0	0	3	91.6 ± 0.7
RSO-PUA/20% HEA	77	20	0	0	3	91.8 ± 0.8
RSO-PUA/30% HEA	67	30	0	0	3	91.8 ± 0.1
RSO-PUA/40% HEA	57	40	0	0	3	93.0 ± 0.3
RSO-PUA/40% TEGDA	57	0	40	0	3	95.0 ± 0.9
RSO-PUA/40% TMPTA	57	0	0	40	3	98.5 ± 1.4

**Table 2:** Thermal properties of the cured RSO-PUA resins.

Samples	*E′*_25_ ^a^ (GPa)	*T*_g_ ^b^ (°C)	*ν*_e_ ^c^ (10^3^ mol/m^3^)	*T*_5_ ^d^ (°C)	*T*_10_ ^e^ (°C)	*T*_max_ ^f^ (°C)	*w*_char_ ^g^ (%)
RSO-PUA	1.5	89.3	168.4	241.9	266.9	431.5	0.8
RSO-PUA/10% HEA	1.6	82.5	184.3	221.9	266.9	422.0	4.4
RSO-PUA/20% HEA	1.3	78.6	144.4	219.4	264.4	418.0	4.9
RSO-PUA/30% HEA	1.4	69.3	163.2	204.2	259.2	421.8	4.5
RSO-PUA/40% HEA	0.9	57.7	105.2	229.1	271.6	425.3	2.6
RSO-PUA/40% TEGDA	1.7	74.8	194.4	246.6	279.1	420.6	4.9
RSO-PUA/40% TMPTA	2.3	109.4	241.2	261.6	291.6	437.1	5.7

^a^ Storage modulus at 25 °C. ^b^ Glass transition temperature. ^c^ Crosslink density. ^d^ 5% weight loss temperature. ^e^ 10% weight loss temperature. ^f^ Maximum decomposition temperature. ^g^ Char yield.

**Table 3:** Mechanical properties and coating properties of the cured RSO-PUA resins.

Samples	*σ*^a^ (MPa)	*E*^b^ (MPa)	*ε*^c^ (%)	*Ad.* ^d^	*P.H.* ^e^	*Fl.*^f^ (mm)
RSO-PUA	11.2 ± 2.6	314.8 ± 65.4	5.2 ± 0.2	2	2H	2
RSO-PUA/10% HEA	16.3 ± 0.6	330.6 ± 42.2	8.2 ± 0.7	2	2H	2
RSO-PUA/20% HEA	16.2 ± 1.0	354.1 ± 38.9	6.7 ± 1.7	2	H	2
RSO-PUA/30% HEA	10.2 ± 0.3	251.0 ± 14.3	11.4 ± 1.8	2	2H	2
RSO-PUA/40% HEA	5.4 ± 0.8	113.3 ± 16.0	39.8 ± 5.4	2	3H	2
RSO-PUA/40% TEGDA	12.5 ± 0.9	304.0 ± 32.4	6.5 ± 1.0	7	3H	18
RSO-PUA/40% TMPTA	11.7 ± 0.9	443.6 ± 0.4	6.7 ± 1.3	2	3H	2

^a^ Tensile strength; ^b^ Tensile modulus; ^c^ Elongation at break. ^d^ Adhesion of coatings. ^e^ Pencil hardness of coatings. ^f^ Flexibility of coatings.

**Table 4:** Swelling properties and UV-curing behaviors of the cured RSO-PUA resins.

Samples	Swelling (%)				α_f_ ^a^ (%)	R_p_ ^b^ (s^−1^)
	Water	Ethanol	Acetone	Toluene		
RSO-PUA	0.22	2.87	6.71	5.52	65.5	5.5
RSO-PUA/10% HEA	0.49	1.42	6.45	5.04	84.5	15.8
RSO-PUA/20% HEA	0.35	2.24	7.50	2.56	87.0	15.3
RSO-PUA/30% HEA	0.35	3.25	7.67	3.23	97.4	22.1
RSO-PUA/40% HEA	0.38	1.96	4.06	2.51	93.1	20.5
RSO-PUA/40% TEGDA	0.48	1.14	2.17	0.29	66.4	10.0
RSO-PUA/40% TMPTA	0.27	0.78	0.93	0.48	70.3	12.1

^a^ Final C=C conversion; ^b^ Maximum C=C conversion rate.

**Table 5:** The printing parameters of RSO-PUA/HEA40% resin and commercial resin.

Sample	Penetration Depth (mm)	Critical Cure Time (s)	Critical Exposure Energy (mJ/cm^2^)	*R* ^2^
RSO-PUA/HEA40%	0.327	1.90	15.20	0.99
Commercial resin	0.314	2.04	16.32	0.98

## Data Availability

Not applicable.

## Supplementary Materials

The following are available online, Figure S1: Chemical structures of the adopted diluents, Figure S2: FT-IR spectra of IPDI-HEA, Figure S3: ^1^H NMR spectra of RSO, Figure S4: The fractured surface SEM images of the UV-cured RSO-PUA resins (Magnification: 1000).

## Sample Availability

Samples of the compounds are not available from the authors.
