# Supplementary material for: Rubber Seed Oil-Based UV-Curable Polyurethane Acrylate Resins for Digital Light Processing (DLP) 3D Printing
Source: Molecules. 2021 Sep 8;26(18):5455. doi: 10.3390/molecules26185455 (PMC8469773; doi:10.3390/molecules26185455)
Supplement: Supplementary file 1 [file molecules-26-05455-s001.zip › molecules-1349307-supplementary.pdf]

Supporting Information (SI)

Rubber seed oil-based UV-curable polyurethane acrylate resins for digital light processing (DLP) 3D printing

Yun Hu [1,2], Guoqiang Zhu [1,2], Jinshuai Zhang [1,2], Jia Huang [1,2], Xixi Yu [1,2], Qianqian Shang [1,2], Rongrong An [3], Chengguo Liu [1,2,*], Lihong Hu [1,2] and Yonghong Zhou [1,2]

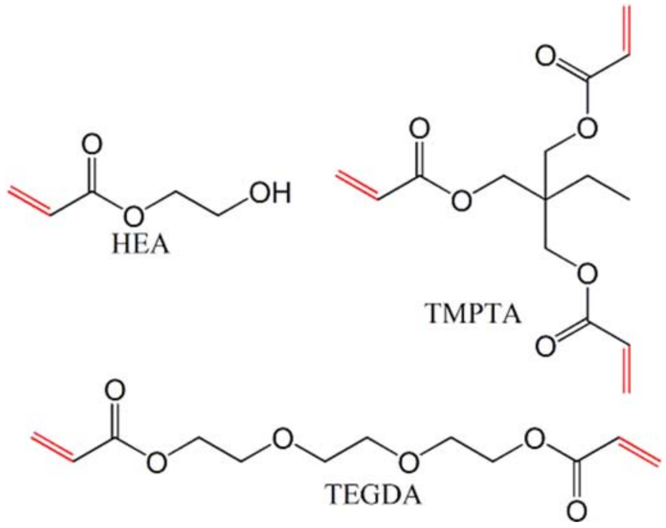

Figure S1. Chemical structures of the adopted diluents

Figure S2 shows the FT-IR spectrum of IPDI-HEA. The strong absorption peak at 2265 cm^{-1} represented the -NCO vibrations from IPDI and the peak at 1719 cm^{-1} was ascribed to the C=O groups. The peak at 1638 cm^{-1} was attributed to C=C stretching vibration absorption.

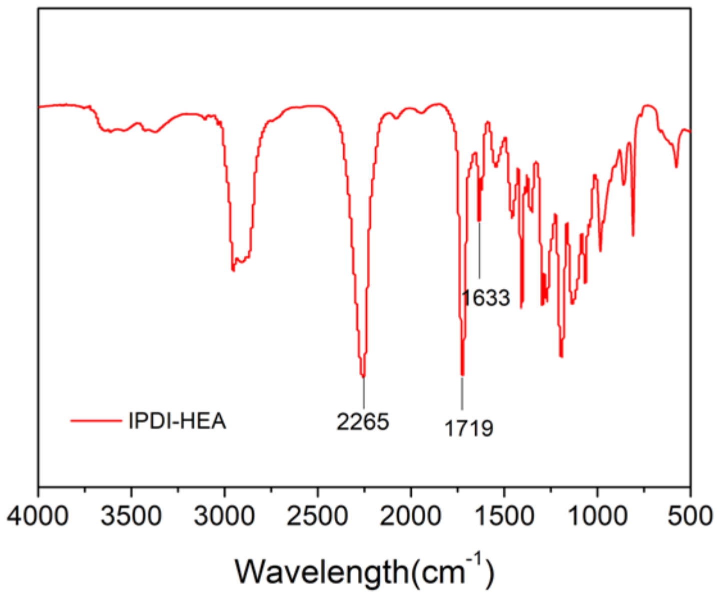

Figure S2. FT-IR spectra of IPDI-HEA

Figure S3 shows the ^1H NMR spectrum of RSO. The peak at 0.8−1.0 ppm was assigned to the terminal $-CH_3$, the peak at 1.2−2.3 ppm attributed to the $-CH_2-CH_2-CH_2-$ long chain. The chemical shift of $-CH_2-$ and −CH− of the glycerol moiety were observed at 4.1–4.3 ppm and 5.3 ppm, respectively. The peaks at 5.3–5.4 ppm represented the protons on the carbon-carbon double bonds of fatty acid chains.

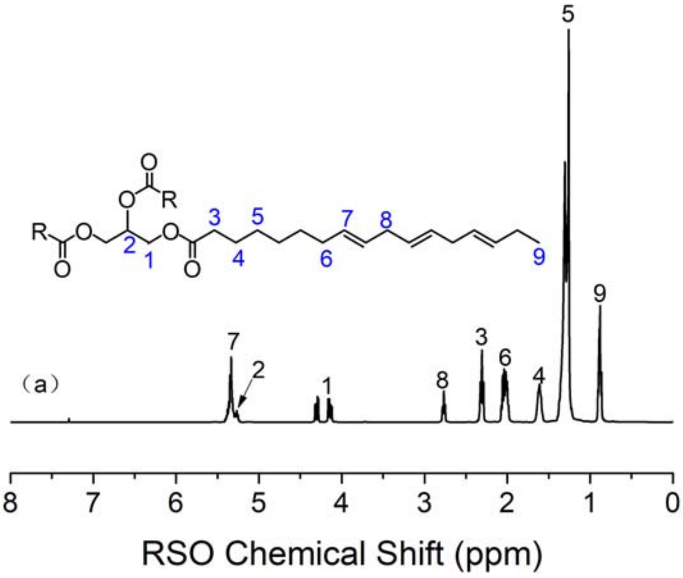

Figure S3. ^{1}H NMR spectra of RSO

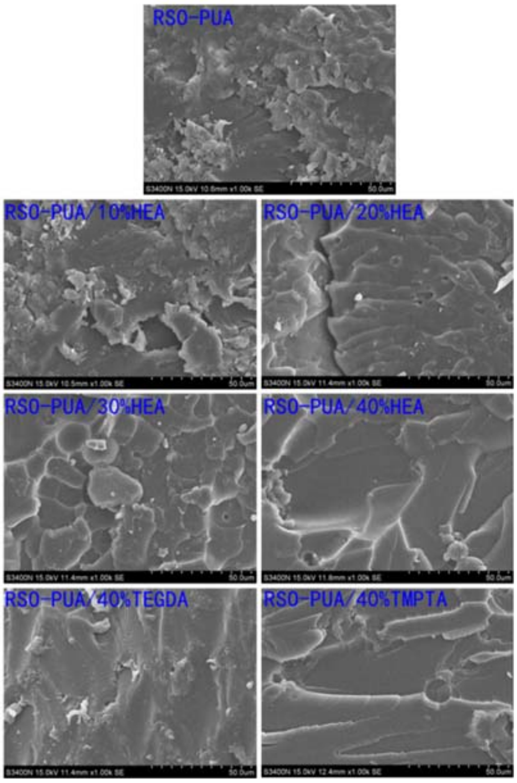

Figure S4. The fractured surface SEM images of the UV-cured RSO-PUA resins (Magnification: 1000).
